# The Value of Laparoscopic Simultaneous Colorectal and Hepatic Resection for Synchronous Colorectal Cancer Liver Metastasis: A Propensity Score Matching Study

**DOI:** 10.3389/fonc.2022.916455

**Published:** 2022-07-12

**Authors:** Jiamin Zhou, Longhai Feng, Xinxiang Li, Miao Wang, Yiming Zhao, Ning Zhang, Longrong Wang, Ti Zhang, Anrong Mao, Ye Xu, Lu Wang

**Affiliations:** ^1^ Department of Hepatic Surgery, Shanghai Cancer Center, Fudan University, Shanghai, China; ^2^ Department of Oncology, Shanghai Medical College, Fudan University, Shanghai, China; ^3^ Department of Colorectal Surgery, Shanghai Cancer Center, Fudan University, Shanghai, China

**Keywords:** colorectal cancer, synchronous liver metastasis, laparoscopy, simultaneous resection, prognosis

## Abstract

**Purpose:**

The aim of this study is to investigate the value of total laparoscopic simultaneous colorectal and hepatic resection in patients with synchronous colorectal cancer liver metastases (sCRLMs).

**Methods:**

sCRLM patients who underwent simultaneous resection from December 2014 to December 2018 in Shanghai Cancer Center, Fudan University were recruited and analyzed retrospectively. The patients were divided into laparoscopic, open, and hybrid surgery groups. The intraoperative information, postoperative short-term outcome, and long-term survival were compared among the three groups. Propensity score matching (PSM) was performed to balance baselines.

**Results:**

A total of 281 patients were recruited. After PSM, 34 patients were selected from both the laparoscopic and the open surgery group. Forty-seven patients were also selected from both the laparoscopic and the hybrid surgery group. The clinicopathologic baselines between the laparoscopic surgery group and the other two groups were well matched. All the operation-related indicators between laparoscopic surgery and hybrid surgery were similar. However, compared with open surgery, laparoscopic surgery showed significantly longer operation time (229.09 ± 10.94 min vs. 192.24 ± 9.49 min, *p* = 0.013) and less intraoperative blood loss [100.00 (50.00–300.00) ml vs. 200.00 (150.00–400.00) ml, *p* = 0.021]. For postoperative morbidity, there was no significant difference between the laparoscopic surgery group and the hybrid or the open surgery group (23.40% vs. 31.91% and 17.65% vs. 26.47%, *p* = 0.356 and *p* = 0.380). Long-term survival analysis showed that there were no significant differences in all 1-, 3-, and 5-year overall survival, liver recurrence-free survival (RFS), and whole RFS between laparoscopic surgery and hybrid surgery (*p* = 0.334, *p* = 0.286, and *p* = 0.558) or open surgery (*p* = 0.230, *p* = 0.348, and *p* = 0.450).

**Conclusions:**

Laparoscopic simultaneous resection for sCRLM shows slight advantages in surgical safety and short-term outcome, and does not compromise long-term survival.

## Introduction

Colorectal cancer (CRC) is one of the most common tumors in the digestive system. Both the number of estimated new cases and deaths of CRC rank third among all cancers (1). Liver is the most common metastatic site of CRC and 15%–25% of CRC patients are found to be accompanied by liver metastasis at first diagnosis, called synchronous colorectal cancer liver metastasis (sCRLM) (2, 3). There are two different options for surgical resection of primary and metastatic tumors: simultaneous resection and staged resection for sCRLM (primary first or liver first). Recently, a large prospective randomized controlled trial (RCT) demonstrated that in spite of no significant difference, the long-term survival benefit of patients who underwent simultaneous resection seemed to be better compared with staged resection and the morbidity was similar (4). Furthermore, the lower treatment cost makes simultaneous resection more advantageous (5). Therefore, although the short- and long-term benefits of the above treatment options are still controversial (4, 6–8), simultaneous resection is generally accepted to treat appropriate sCRLM patients in the current clinical practice.

Laparoscopic surgery has been widely used in the field of hepatobiliary and colorectal surgery for more than 20 years. Especially in CRLM, its safety and effectiveness have been confirmed in the resection of primary tumor and liver metastases, respectively (9–12). On this basis, it is speculated that the laparoscopic approach for simultaneous colorectal and hepatic resection can alleviate the disadvantage of excessive trauma moderately. At the same time, the dilemma of surgical incision selection may be solved, and even long-term survival benefits are expected. At present, total laparoscopic simultaneous colorectal and hepatic resection for sCRLM patients is gradually accepted, but its short-term and long-term results seem to be less than expected (13–15). Therefore, in order to explore the therapeutic value of total laparoscopic simultaneous resection for sCRLM, this study retrospectively analyzed the data of patients with sCRLM in our center, and propensity score matching (PSM) was performed to reduce bias in patient selection and make the results more convincing.

## Methods

### Study Design

**Figure 1:**
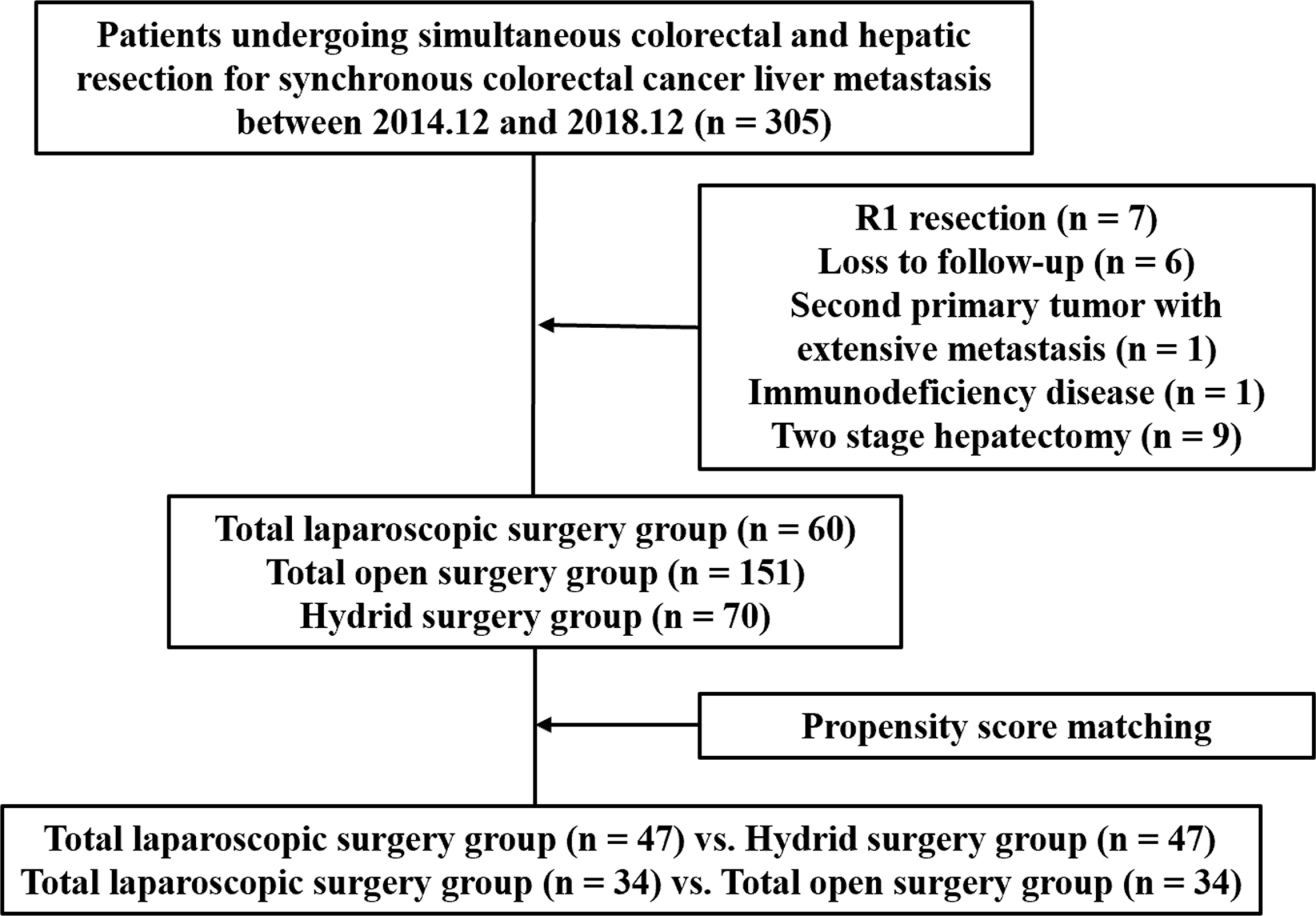
The flowchart of this study.

From December 2014 to December 2018, 305 consecutive sCRLM patients who underwent simultaneous primary and hepatic resection in Shanghai Cancer Center, Fudan University were recruited retrospectively. Inclusion criteria were patients with pathologically proven CRLM who underwent simultaneous resection. Exclusion criteria were as follows: (1) R1 resection, (2) loss to follow-up, (3) second primary tumor with extensive metastasis, (4) immunodeficiency disease, and (5) two-stage hepatectomy. Eventually, 281 patients were recruited in a further study (**Figure 1**). These patients were grouped according to the surgical approaches. Both primary CRC and liver metastases resected by the laparoscopic approach were included in the total laparoscopic surgery group. Both primary CRC and liver metastases resected by the open approach were included in the total open surgery group. Primary CRC and metastatic lesions resected by different surgical approaches were included in the hybrid surgery group. All surgical procedures were performed by the experienced teams from the Department of Hepatic Surgery and the Department of Colorectal Surgery. This study was approved by the Research Ethics Committee of our center.

### Follow-Up Procedures

As follow-up procedures, abdominal enhanced CT scanning or MRI, serum CEA levels, and chest radiographs were monitored with an interval of 3 months after hepatectomy. Overall survival (OS) was defined as the interval between surgery and death. Recurrence-free survival (RFS) was defined as the interval between hepatectomy and recurrence. Liver RFS was limited in the liver recurrence. Whole RFS was defined as the interval between hepatectomy and any site recurrence in the patients without extrahepatic diseases. The median follow-up time was 47 months.

### Observation Variables

Age, gender, body mass index (BMI), preoperative CEA level, extrahepatic disease, and features of liver metastasis (number, size and distribution) were routinely collected. T stage and N stage were defined according to AJCC criteria (8th edition). T0 in this study was identified as T stage could not be verified due to the great response to preoperative treatment. Primary tumors located in the cecum, ascending colon, and transverse colon were classified as right-sided CRC. The splenic flexure, descending colon, sigmoid colon, and rectum were classified as left-sided CRC. Postoperative complications were defined according to the Clavien–Dindo classification criteria.

### Statistical Analysis

PSM analysis was performed in this study to reduce bias using SPSS 22.0 for Windows (SPSS Inc, Chicago, IL). The variables in the clinicopathological baseline that were not balanced and might affect the results were included in the calculation. The variables to be balanced between the laparoscopic and the hybrid surgery group included liver metastasis distribution, number of liver metastasis, and proportion of preoperative chemotherapy. The variables to be balanced between the laparoscopic and the open surgery group included liver metastasis distribution, number of liver metastasis, primary tumor site, and proportion of preoperative chemotherapy. Two sub-groups in the hybrid surgery group, laparoscopic primary CRC resection combined with open liver metastases resection (Lap CRC/Open LM) and open primary CRC resection combined with laparoscopic LM resection (Open CRC/Lap LM), were also balanced by variables including age, liver metastasis distribution, number of liver metastasis, and proportion of preoperative chemotherapy. The propensity score was generated using logistic regression with these variables, and the caliper value was set as 0.02. The patients were selected using nearest-neighbor matching without replacement at a ratio of 1:1. Two-sample Student’s *t*-test or Mann–Whitney *U* test was performed to compare quantitative variables. For data analyzed with the two-sample Student’s *t*-test, the data were presented as mean ± standard error, and for data analyzed with the Mann–Whitney *U* test, the data were presented as median (interquartile range). Pearson *χ* ^2^ test or Fisher’s exact test was performed to compare qualitative variables. Statistical analysis was performed using SPSS 22.0. *p* < 0.05 was considered statistically significant.

## Results

In total, 281 patients were eligible for the analysis. There were 60 patients who underwent total laparoscopic surgery, 151 patients who underwent total open surgery, and 70 patients who underwent hybrid surgery. There was a higher proportion of the Lap CRC/Open LM sub-group in the hybrid surgery group (68.57% vs. 31.43%). The clinicopathologic characteristics of these cohorts and sub-groups are summarized in **Table 1** and **Supplementary Table 1**. Compared with the hybrid surgery group, there was a lower proportion of patients with liver metastasis bilobar distribution (23.33% vs. 42.86%, *p* = 0.019), and a higher proportion of patients who underwent preoperative chemotherapy (60.00% vs. 32.86%, *p* = 0.002) and patients with less liver metastasis [1.00 (1.00–2.00) vs. 2.00 (1.00–4.00), *p* = 0.009] in the total laparoscopic surgery group. Compared with the total open surgery group, the total laparoscopic surgery group demonstrated the following features: a lower proportion of patients with liver metastasis bilobar distribution (23.33% vs. 56.29%, *p* < 0.001), primary right-sided CRC (16.67% vs. 67.55%, *p* < 0.001), and braf gene mutation (0.00% vs. 5.88%, *p* = 0.030), and a higher proportion of patients who underwent preoperative chemotherapy (60.00% vs. 39.74%, *p* = 0.008) and patients with less liver metastasis [1.00 (1.00–2.00) vs. 2.00 (1.00–5.00), *p* < 0.001]. In the hybrid surgery group, the patients were younger [54.50 (47.25–62.75) years vs. 61.50 (58.00–66.00) years, *p* = 0.008], the patients had more liver metastasis [3.00 (1.25–5.00) vs. 1.00 (1.00–2.00), *p* = 0.001], and there was a higher proportion of patients with liver metastasis bilobar distribution (56.25% vs. 13.64%, *p* = 0.001) and of patients who underwent preoperative chemotherapy (75.00% vs. 50.00%, *p* = 0.039) in the Lap CRC/Open LM sub-group. The remaining clinicopathologic characteristics were balanced among these groups.

**Table 1:** The clinicopathological characteristics of patients.

Characteristic	Before propensity score matching					After propensity score matching					
	Laparoscopy(*n* = 60)	Hybrid (*n* = 70)	*p*	Open (*n* = 151)	*p*	Laparoscopy(*n* = 47)	Hybrid(*n* = 47)	*p*	Laparoscopy (*n* = 34)	Open (*n* = 34)	*p*
**Age, years**	61.00 (53.00–66.00)	57.50 (49.00–64.00)	0.069	60.00 (51.00–66.00)	0.446	60.00 (53.00–64.00)	57.00 (49.00–63.00)	0.224	59.47 ± 1.51	58.62 ± 1.49	0.689
**Gender, %**			0.758		0.590			1.000			1.000
**Male**	37 (61.67)	45 (64.29)		87 (57.62)		31 (65.96)	31 (65.96)		20 (58.82)	20 (58.82)	
**Female**	23 (38.33)	25 (35.71)		64 (42.38)		16 (34.04)	16 (34.04)		14 (41.18)	14 (41.18)	
**Body mass index**	23.02 (21.36–25.60)	22.94 (20.57–24.88)	0.533	23.05 (20.90–25.56)	0.925	23.29 ± 0.43	23.03 ± 0.44	0.663	22.90 ± 0.49	23.38 ± 0.48	0.487
**Preoperative CEA level, ng/ml**	10.76 (4.43–42.75)	7.86 (2.99–29.14)	0.337	11.19 (4.10–39.53)	0.995	8.68 (4.34–24.30)	11.06 (4.36–35.00)	0.563	12.93 (4.31–50.62)	10.48 (4.96–47.70)	0.917
**Extrahepatic disease, %**			0.936		0.561			0.765			1.000
**Yes**	8 (13.33)	9 (12.86)		25 (16.56)		6 (12.77)	7 (14.89)		5 (14.71)	5 (14.71)	
**No**	52 (86.67)	61 (57.14)		126 (83.44)		41 (87.23)	40 (85.11)		29 (85.29)	29 (85.29)	
**Liver metastasis distribution, %**			**0.019**		**<0.001**			0.797			0.622
**Unilobar**	46 (76.67)	40 (57.14)		66 (43.71)		37 (78.72)	38 (80.85)		13 (38.24)	15 (44.12)	
**Bilobar**	14 (23.33)	30 (42.86)		85 (56.29)		10 (21.28)	9 (19.15)		21 (61.76)	19 (55.88)	
**Number of liver metastasis**	1.00 (1.00–2.00)	2.00 (1.00–4.00)	**0.009**	2.00 (1.00–5.00)	**<0.001**	1.00 (1.00–2.00)	1.00 (1.00–3.00)	0.731	1.00 (1.00–2.25)	2.00 (1.00–3.25)	0.120
**Size of largest liver metastasis, cm**	2.35 (1.50–3.00)	2.50 (1.65–3.78)	0.400	2.80 (1.50–4.00)	0.252	2.00 (1.50–3.00)	2.50 (1.70–4.50)	0.218	2.35 (1.43–3.00)	2.50 (1.28–3.50)	0.990
**Primary tumor site, %**			0.090		**<0.001**			1.000			0.582
**Left-sided**	50 (83.33)	65 (92.86)		49 (32.45)		42 (89.36)	42 (89.36)		24 (70.59)	26 (76.47)	
**Right-sided**	10 (16.67)	5 (7.14)		102 (67.55)		5 (10.64)	5 (10.64)		10 (29.41)	8 (23.53)	
**Primary T stage, %**			0.575		0.191			0.877			0.810
**T0**	0 (0.00)	1 (14.29)		2 (1.32)		–	–		–	–	
**T1**	2 (3.33)	0 (0.00)		0 (0.00)		1 (2.13)	0 (0.00)		1 (2.94)	0 (0.00)	
**T2**	5 (8.33)	5 (7.14)		10 (6.62)		4 (8.51)	4 (8.51)		2 (5.88)	1 (2.94)	
**T3**	44 (73.33)	55 (78.57)		107 (70.86)		34 (72.34)	37 (78.72)		26 (76.47)	26 (76.47)	
**T4**	9 (15.00)	9 (12.86)		32 (21.19)		8 (17.02)	6 (12.77)		5 (14.71)	7 (20.59)	
**Primary N stage, %**			0.592		0.933			0.242			0.486
**N0**	18 (30.00)	20 (28.57)		45 (29.80)		12 (25.53)	13 (27.66)		9 (26.47)	5 (14.71)	
**N1**	31 (51.67)	32 (45.71)		75 (49.67)		28 (59.57)	21 (44.68)		17 (50.00)	20 (58.82)	
**N2**	11 (18.33)	18 (25.71)		31 (20.53)		7 (14.89)	13 (27.66)		8 (23.53)	9 (26.47)	
**ras gene status, %**			0.718		0.154			0.598			0.780
**Wild type**	25 (54.35)	33 (57.89)		50 (42.02)		20 (52.63)	24 (58.54)		11 (42.31)	12 (46.15)	
**Mutation**	21 (45.65)	24 (42.11)		69 (57.98)		18 (47.37)	17 (41.46)		15 (57.69)	14 (53.85)	
**braf gene status, %**			1.000		**0.030**			1.000			1.000
**Wild type**	46 (100.00)	57 (100.00)		112 (94.12)		38 (100.00)	41 (100.00)		26 (100.00)	25 (96.15)	
**Mutation**	0 (0.00)	0 (0.00)		7 (5.88)		0 (0.00)	0 (0.00)		0 (0.00)	1 (3.85)	
**Preoperative chemotherapy, %**			**0.002**		**0.008**			0.836			1.000
**Yes**	24 (40.00)	47 (67.14)		91 (60.26)		24 (51.06)	25 (53.19)		14 (41.18)	14 (41.18)	
**No**	36 (60.00)	23 (32.86)		60 (39.74)		23 (48.94)	22 (46.81)		20 (58.82)	20 (58.82)	

p < 0.05 was considered statistically significant and shown in bold.

The operative results and postoperative outcomes in these three cohorts are displayed in **Table 2**. Compared with the total open surgery group, the laparoscopic surgery group showed significantly longer operation time (229.25 ± 8.27 min vs. 200.48 ± 4.68 min, *p* = 0.002), less intraoperative blood loss [150.00 (100.00–300.00) ml vs. 200.00 (150.00–400.00) ml, *p* < 0.001], lower proportion of intraoperative transfusion (11.67% vs. 24.50%, *p* = 0.038), earlier initial defecation time [3.00 (2.00–5.00) days vs. 4.00 (3.00–6.00) days, *p* < 0.001], and less postoperative hospital stay [8.00 (7.00–11.00) days vs. 9.00 (8.00–12.00) days, *p* = 0.005]. However, when compared with the hybrid surgery group, only less intraoperative blood loss [150.00 (100.00–300.00) ml vs. 200.00 (100.00–400.00) ml, *p* = 0.036] was observed in the laparoscopic surgery group. For postoperative morbidity, there was no significant difference between the laparoscopic surgery group and the other two groups (25.00% vs. 31.43% and 35.76%, *p* = 0.418 and *p* = 0.078). In the hybrid surgery group, the Lap CRC/Open LM sub-group demonstrated longer operation time [236.50 (194.25–275.75) min vs. 170.00 (148.00–239.75) min, *p* = 0.006] and more postoperative hospital stay [10.00 (8.00–12.00) days vs. 8.50 (7.00–10.00) days, *p* = 0.025] compared with the Open CRC/Lap LM sub-group (**Supplementary Table 2**).

**Table 2:** Operation-related factors.

Characteristic	Before propensity score matching					After propensity score matching					
	Laparoscopy(*n* = 60)	Hybrid (*n* = 70)	*p*	Open (*n* = 151)	*p*	Laparoscopy(*n* = 47)	Hybrid (*n* = 47)	*p*	Laparoscopy(*n* = 34)	Open (*n* = 34)	*p*
**Operation time, min**	229.25 ± 8.27	228.01 ± 8.80	0.920	200.48 ± 4.68	**0.002**	232.53 ± 10.01	218.94 ± 44.16	0.375	229.09 ± 10.94	192.24 ± 9.49	**0.013**
**Intraoperative blood loss, ml**	150.00 (100.00–300.00)	200.00 (100.00–400.00)	**0.036**	200.00 (150.00–400.00)	**<0.001**	150.00 (100.00–300.00)	200.00 (100.00–400.00)	0.290	100.00 (50.00–300.00)	200.00 (150.00–400.00)	**0.021**
**Intraoperative transfusion, %**			0.139		**0.038**			0.180			1.000
**Yes**	7 (11.67)	15 (21.43)		37 (24.50)		6 (12.77)	11 (23.40)		5 (14.71)	5 (14.71)	
**No**	53 (88.33)	55 (78.57)		114 (75.50)		41 (87.23)	36 (76.60)		29 (85.29)	29 (85.29)	
**Initial defecation time, days**	3.00 (2.00–5.00)	4.00 (3.00–5.00)	0.412	4.00 (3.00–6.00)	**<0.001**	3.00 (2.00–5.00)	4.00 (3.00–5.00)	0.348	4.00 (2.00–6.00)	4.00 (3.00–5.00)	0.709
**Postoperative hospital stay, days**	8.00 (7.00–11.00)	9.00 (7.75–12.00)	0.092	9.00 (8.00–12.00)	**0.005**	8.00 (7.00–11.00)	10.00 (8.00–12.00)	0.095	8.00 (7.00–11.00)	9.00 (8.00–12.25)	0.052
**Postoperative complications, %**			0.468		0.415			0.285			0.434
**Absent**	45 (75.00)	48 (68.57)		94 (64.24)		36 (76.60)	32 (68.09)		28 (82.35)	25 (73.53)	
**Grade I**	8 (13.33)	8 (11.43)		27 (17.88)		6 (12.77)	4 (8.51)		1 (2.94)	3 (8.82)	
**Grade II**	4 (6.67)	8 (11.43)		11 (7.28)		3 (6.38)	7 (14.89)		3 (8.82)	1 (2.94)	
**Grade III**	2 (3.33)	6 (8.57)		15 (9.93)		1 (2.13)	4 (8.51)		2 (5.88)	3 (8.82)	
**Grade IV**	1 (1.67)	0 (0.00)		4 (2.65)		1 (2.13)	0 (0.00)		0 (0.00)	2 (5.88)	

p < 0.05 was considered statistically significant and shown in bold.

Long-term survival analysis showed that in the total laparoscopic surgery group, 1-, 3-, and 5-year OS were 97%, 72%, and 52%, respectively; 1-, 3- and 5-year liver RFS were 75%, 58%, and 51%, respectively; and 1-, 3-, and 5-year whole RFS were 73%, 42%, and 32%, respectively. Compared with the open surgery group, OS and whole RFS were similar (*p* = 0.122 and *p* = 0.172), and liver RFS was significantly better (60%, 39%, and 37%, *p* = 0.027). Compared with the hybrid surgery group, OS and whole RFS were similar (*p* = 0.757 and *p* = 0.062), and liver RFS was also significantly better (56%, 40%, and 34%, *p* = 0.048).

**Figure 2:**
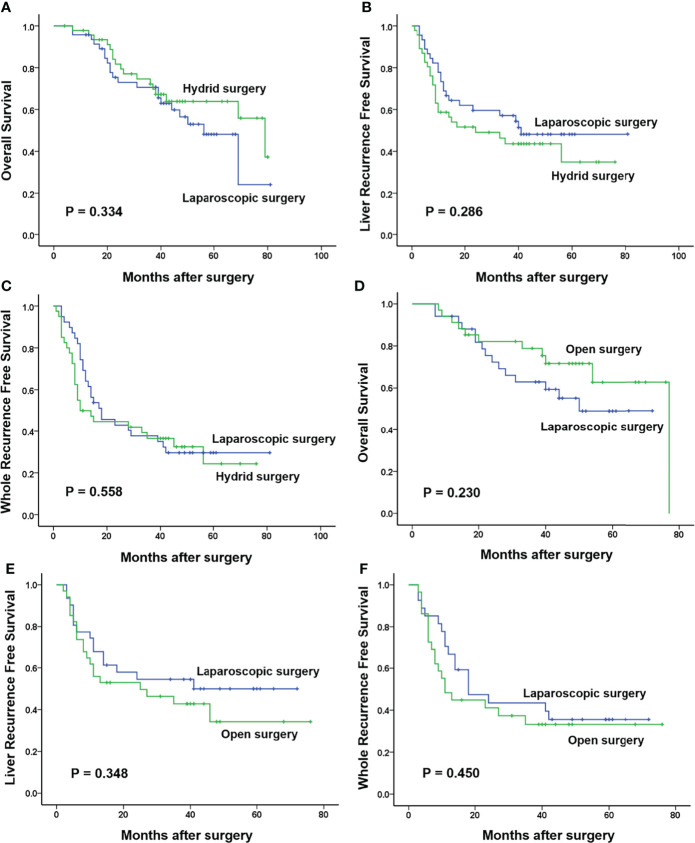
Long-term survival in three groups after propensity score matching. **(A–C)** One-, 3-, and 5-year overall survival (OS), liver recurrence-free survival (RFS), and whole RFS in the laparoscopic surgery group were 96%, 71%, and 48%; 73%, 57%, and 47%; and 69%, 38%, and 29%, respectively, which were similar with those in the hybrid surgery group (98%, 75%, and 63%; 58%, 43%, and 37%; and 49%, 36%, and 27%, respectively; *p* = 0.334, *p* = 0.286, and *p* = 0.558). **(D–F)** There were also no significant differences in 1-, 3-, and 5-year OS, liver RFS, and whole RFS between the laparoscopic surgery group (94%, 63%, and 48%; 68%, 54%, and 50%; and 70%, 44%, and 35%, respectively) and the open surgery group (94%, 79%, and 63%; 56%, 43%, and 38%; and 48%, 33%, and 33%, respectively; *p* = 0.230, *p* = 0.348, and *p* = 0.450).

After PSM, 34 patients were selected from both the laparoscopic surgery group and the open surgery group and 47 patients were also selected from both the laparoscopic surgery group and the hybrid surgery group for further analysis. The clinicopathologic baselines between the laparoscopic surgery group and the other two groups were well matched (**Table 1**). There were also more balanced clinicopathologic characteristics of the two sub-groups in the hybrid surgery group (both 11 selected patients; **Supplementary Table 1**). All the operation-related indicators in the laparoscopic surgery and hybrid surgery group were similar. However, compared with the open surgery group, the laparoscopic surgery group showed significantly longer operation time (229.09 ± 10.94 min vs. 192.24 ± 9.49 min, *p* = 0.013) and significantly less intraoperative blood loss [100.00 (50.00–300.00) ml vs. 200.00 (150.00–400.00) ml, *p* = 0.021]. In spite of no significance, the postoperative hospital stay of the laparoscopic surgery group tended to be shorter than the other two groups (*p* = 0.095 and *p* = 0.052). For postoperative morbidity, there was no significant difference between the laparoscopic surgery group and the hybrid or the open surgery group (23.40% vs. 31.91% and 17.65% vs. 26.47%, *p* = 0.356 and *p* = 0.380; **Table 2**). In the hybrid surgery group, the Lap CRC/Open LM sub-group only showed more intraoperative blood loss than the other sub-group [200.00 (200.00–500.00) ml vs. 100.00 (100.00–200.00) ml, *p* = 0.005; **Supplementary Table 2**]. Long-term survival analysis showed that there were no significant differences in all 1-, 3-, and 5-year OS, and liver RFS and whole RFS between the laparoscopic surgery group and the hybrid group (*p* = 0.334, *p* = 0.286, and *p* = 0.558) or open surgery group (*p* = 0.230, *p* = 0.348, and *p* = 0.450) (**Figure 2**).

## Discussion

Laparoscopic surgery has been gradually applied to simultaneous colorectal and hepatic resection for the patients with sCRLM in the past decade (16, 17), and its safety and effectiveness were initially confirmed in several small-scale studies and case reports (18–21). Compared with hybrid surgery and total open surgery, total laparoscopic simultaneous resection is considered to be characterized by sophisticated operation, small incision, and rapid postoperative recovery (22). After 2014, related studies were gradually increasing. In these previous studies, advances in perioperative safety and short-term outcomes, including intraoperative blood loss, postoperative hospital stay, and postoperative morbidity, were observed in the total laparoscopic simultaneous resection approach (15, 18, 23–27). Although these advantages seemed unstable and could not be observed in all studies (14), both long-term OS and RFS were not compromised. Compared with two recent studies (26, 27), three large-scale cohorts were recruited in our study, and these patients were treated in a relatively short period in recent years, which were in accordance with the present treatment guideline of sCRLM. In addition to the well-matched baseline, and due to the application of PSM, it was the first time that the short- and long-term outcomes between total laparoscopic surgery and hybrid surgery were contrasted, and no significant difference was revealed. On the other hand, except for postoperative morbidity, the comparison between total laparoscopic surgery and total open surgery confirmed the results from the meta-analysis of Pan et al. (28) recently.

It has been proved that the long-term prognosis of patients with right-sided CRC is worse than that of left-sided CRC (29, 30). Although the incidence of liver metastasis is higher in the patients with left-sided CRC (28.4% vs. 22.1%), the liver metastases from right-sided CRC are associated with a higher rate of ras mutation and a wider distribution of liver metastasis lesions, which eventually lead to a poorer prognosis (31–33). In clinical practice, due to the close anatomical structure between the primary right-sided CRC and liver metastasis focus, simultaneous colorectal and hepatic resection could be performed under the same surgical incision (inverted L-shaped incision), prompting the majority of patients with primary right-sided sCRLM to undergo simultaneous total open resection. This is different from primary left-sided sCRLM, which entails a slightly difficult selection of surgical incision. This tendency has also been shown in our study. Therefore, the total open surgery group showed significantly worse short- and long-term outcomes compared with the total laparoscopic surgery group before PSM.

Based on the literature review and the results of our study, it could be confirmed that total laparoscopic surgery could bring certain surgical safety and short-term outcome benefits to patients without compromising long-term survival. Compared with hybrid surgery, total laparoscopic surgery did not show any advantages. Of course, this conclusion should be further confirmed by prospective RCTs. Total laparoscopic resection in suitable patients could be performed prudently in larger medical centers with sufficient experience in laparoscopic colorectal or hepatic surgery after multidisciplinary evaluation.

## Data Availability Statement

The raw data supporting the conclusions of this article will be made available by the authors, without undue reservation.

## Ethics Statement

The studies involving human participants were reviewed and approved by the Institutional Review Board and the Ethics Committee of Shanghai Cancer Center (SCCIR). The patients/participants provided their written informed consent to participate in this study.

## Author Contributions

Study concept and design (JZ, LF, XL, AM, YX, and LW), acquisition of data (JZ, LF, MW, NZ, and LRW), analysis and interpretation of data (JZ, LF, XL, AM, YX, and LW), drafting of the manuscript (JZ, LF, and XL), critical revision of the manuscript for important intellectual content (XL, YZ, TZ, AM, YX, and LW), administrative, technical, or material support (XL, YZ, TZ, AM, YX, and LW), and study supervision (AM, YX, and LW). All authors have made a significant contribution to this study and have approved the final manuscript.

## Funding

This work was supported by the National Science Foundation of China (81874182 and 81902438).

## Conflict of Interest

The authors declare that the research was conducted in the absence of any commercial or financial relationships that could be construed as a potential conflict of interest.

The reviewer JC declared a shared parent affiliation with the authors to the handling editor at the time of review.

## Publisher’s Note

All claims expressed in this article are solely those of the authors and do not necessarily represent those of their affiliated organizations, or those of the publisher, the editors and the reviewers. Any product that may be evaluated in this article, or claim that may be made by its manufacturer, is not guaranteed or endorsed by the publisher.
