# Supplementary material for: The Value of Laparoscopic Simultaneous Colorectal and Hepatic Resection for Synchronous Colorectal Cancer Liver Metastasis: A Propensity Score Matching Study
Source: Front Oncol. 2022 Jul 12;12:916455. doi: 10.3389/fonc.2022.916455 (PMC9315101; doi:10.3389/fonc.2022.916455)
Supplement: Supplementary file 1 [file Table_1.docx]

**Supplementary table 1. The clinicopathological characteristics of patients in hydrid surgery group**

| **Characteristic** | **Before propensity score matching** | | |  | **After propensity score matching** | | |
| --- | --- | --- | --- | --- | --- | --- | --- |
|  | **Lap CRC/Open LM**  **(n = 48)** | **Open CRC/Lap LM**  **(n = 22)** | **P** |  | **Lap CRC/Open LM**  **(n = 11)** | **Open CRC/Lap LM**  **(n = 11)** | **P** |
| **Age, years** | 54.50 (47.25-62.75) | 61.50 (58.00-66.00) | **0.008** |  | 57.64 ± 3.80 | 56.82 ± 2.44 | 0.858 |
| **Gender, %** |  |  | 0.645 |  |  |  | 0.117 |
| **Male** | 30 (62.50) | 15 (68.18) |  |  | 10 (90.91) | 7 (63.64) |  |
| **Female** | 18 (37.50) | 7 (31.82) |  |  | 1 (9.09) | 4 (36.36) |  |
| **Body mass index** | 23.07 ± 0.45 | 22.96 ± 0.66 | 0.890 |  | 22.55 ± 0.75 | 22.77 ± 3.82 | 0.875 |
| **Preoperative CEA level, ng/ml** | 7.48 (2.61-36.22) | 8.48 (4.35-21.57) | 0.970 |  | 5.14 (2.34-11.06) | 5.93 (1.97-12.45) | 0.898 |
| **Extrahepatic disease, %** |  |  | 0.512 |  |  |  | 1.000 |
| **Yes** | 7 (14.58) | 2 (9.09) |  |  | 1 (9.09) | 1 (9.09) |  |
| **No** | 41 (85.42) | 20 (90.91) |  |  | 10 (90.91) | 10 (90.91) |  |
| **Liver metastasis distribution, %** |  |  | **0.001** |  |  |  | 1.000 |
| **Unilobar** | 21 (43.75) | 19 (86.36) |  |  | 8 (72.73) | 8 (72.73) |  |
| **Bilobar** | 27 (56.25) | 3 (13.64) |  |  | 3 (27.27) | 3 (27.27) |  |
| **Number of liver metastasis** | 3.00 (1.25-5.00) | 1.00 (1.00-2.00) | **0.001** |  | 2.00 (1.00-4.00) | 1.00 (1.00-3.00) | 0.478 |
| **Size of largest liver metastasis, cm** | 2.50 (1.70-3.50) | 2.10 (1.50-4.75) | 0.929 |  | 1.50 (1.00-2.50) | 2.50 (1.80-6.00) | 0.088 |
| **Primary tumor site, %** |  |  | 0.674 |  |  |  | 0.085 |
| **Left-sided** | 45 (93.75) | 20 (90.91) |  |  | 11 (100.00) | 9 (81.82) |  |
| **Right-sided** | 3 (6.25) | 2 (9.09) |  |  | 0 (0.00) | 2 (18.18) |  |
| **Primary T stage, %** |  |  | 0.387 |  |  |  | 0.214 |
| **T0** | 1 (2.08) | 0 (0.00) |  |  | 0 (0.00) | 0 (0.00) |  |
| **T1** | 0 (0.00) | 0 (0.00) |  |  | 0 (0.00) | 0 (0.00) |  |
| **T2** | 4 (8.33) | 1 (4.55) |  |  | 3 (27.27) | 0 (0.00) |  |
| **T3** | 39 (81.25) | 16 (72.73) |  |  | 8 (72.73) | 10 (90.91) |  |
| **T4** | 4 (8.33) | 5 (22.73) |  |  | 0 (0.00) | 1 (9.09) |  |
| **Primary N stage, %** |  |  | 0.654 |  |  |  | 0.501 |
| **N0** | 15 (31.25) | 5 (22.73) |  |  | 3 (27.27) | 3 (27.27) |  |
| **N1** | 22 (45.83) | 10 (45.45) |  |  | 5 (45.45) | 7 (63.64) |  |
| **N2** | 11 (21.92) | 7 (31.82) |  |  | 3 (27.27) | 1 (9.09) |  |
| **ras gene status, %** |  |  | 0.451 |  |  |  | 0.142 |
| **Wild type** | 25 (60.98) | 8 (50.00) |  |  | 7 (63.64) | 2 (28.57) |  |
| **Mutation** | 16 (39.02) | 8 (50.00) |  |  | 4 (36.36) | 5 (71.43) |  |
| **braf gene status, %** |  |  | - |  |  |  | - |
| **Wild type** | 41 (100.00) | 16 (100.00) |  |  | 11 (100.00) | 7 (100.00) |  |
| **Mutation** | 0 (0.00) | 0 (0.00) |  |  | 0 (0.00) | 0 (0.00) |  |
| **Preoperative chemotherapy, %** |  |  | **0.039** |  |  |  | 1.000 |
| **Yes** | 36 (75.00) | 11 (50.00) |  |  | 7 (63.64) | 7 (63.64) |  |
| **No** | 12 (25.00) | 11 (50.00) |  |  | 4 (36.36) | 4 (36.36) |  |

CRC: colorectal cancer; Lap CRC/Open LM: laparoscopic primary CRC resection combined with open LM resection; Open CRC/Lap LM: open primary CRC resection combined with laparoscopic LM resection

**Supplementary table 2. Operation related factors in hydrid surgery group**

| **Characteristic** | **Before propensity score matching** | | |  | **After propensity score matching** | | |
| --- | --- | --- | --- | --- | --- | --- | --- |
|  | **Lap CRC/Open LM**  **(n = 48)** | **Open CRC/Lap LM**  **(n = 22)** | **P** |  | **Lap CRC/Open LM**  **(n = 11)** | **Open CRC/Lap LM**  **(n = 11)** | **P** |
| **Operation time, mins** | 236.50 (194.25-275.75) | 170.00 (148.00-239.75) | **0.006** |  | 247.00 (192.00-399.00) | 165.00 (140.00-269.00) | 0.151 |
| **Intraoperative blood loss, ml** | 200.00 (127.50-400.00) | 200.00 (100.00-400.00) | 0.223 |  | 200.00 (200.00-500.00) | 100.00 (100.00-200.00) | **0.005** |
| **Intraoperative transfusion, %** |  |  | 0.650 |  |  |  | 0.335 |
| **Yes** | 11 (22.92) | 4 (18.18) |  |  | 4 (36.36) | 2 (18.18) |  |
| **No** | 37 (77.08) | 18 (81.82) |  |  | 7 (63.64) | 9 (81.82) |  |
| **Initial defecation time, days** | 3.00 (3.00-5.00) | 4.00 (2.00-5.00) | 0.867 |  | 4.18 ± 0.67 | 4.27 ± 0.59 | 0.920 |
| **Postoperative hospital stay, days** | 10.00 (8.00-12.00) | 8.50 (7.00-10.00) | **0.025** |  | 10.64 ± 0.61 | 9.27 ± 0.89 | 0.219 |
| **Postoperative complications, %** |  |  | 0.566 |  |  |  | 1.000 |
| **Absent** | 33 (68.75) | 15 (68.18) |  |  | 8 (72.73) | 8 (72.73) |  |
| **Grade I** | 4 (8.33) | 4 (18.18) |  |  | 1 (9.09) | 1 (9.09) |  |
| **Grade II** | 6 (12.50) | 2 (9.09) |  |  | 1 (9.09) | 1 (9.09) |  |
| **Grade III** | 5 (10.42) | 1 (4.55) |  |  | 1 (9.09) | 1 (9.09) |  |
| **Grade IV** | 0 (0.00) | 0 (0.00) |  |  | 0 (0.00) | 0 (0.00) |  |

CRC: colorectal cancer; LM: liver metastases; Lap CRC/Open LM: laparoscopic primary CRC resection combined with open LM resection; Open CRC/Lap LM: open primary CRC resection combined with laparoscopic LM resection
